# Supplementary material for: Identification of c.1531C>T Pathogenic Variant in the CDH1 Gene as a Novel Germline Mutation of Hereditary Diffuse Gastric Cancer
Source: Int J Mol Sci. 2019 Oct 9;20(20):4980. doi: 10.3390/ijms20204980 (PMC6829381; doi:10.3390/ijms20204980)
Supplement: Supplementary file 1 [file ijms-20-04980-s001.pdf]

International Journal of
Molecular Sciences

MDPI

Supplementary Materials: Identification of the non-synonymous c.1531C>T pathogenic variant in the *CDH1* gene as a novel germline mutation of hereditary diffuse gastric cancer

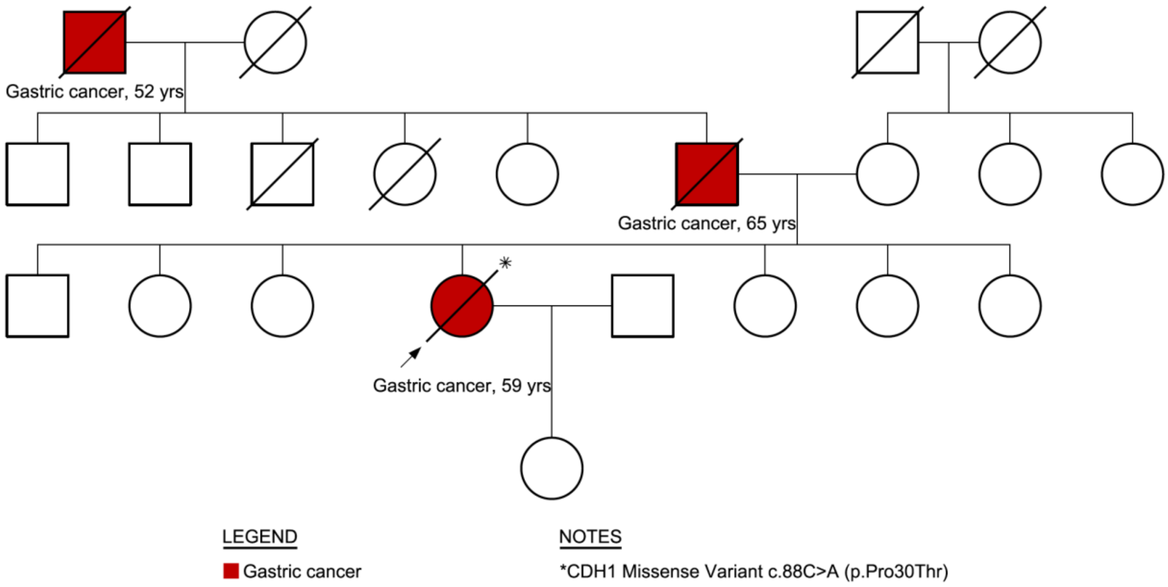

Figure S1. Family pedigree of proband with c88C>A (p.Pro30Thr, rs139866691) variant in a family history of HDGC. In red, the proband and two individuals in which DGC was confirmed.

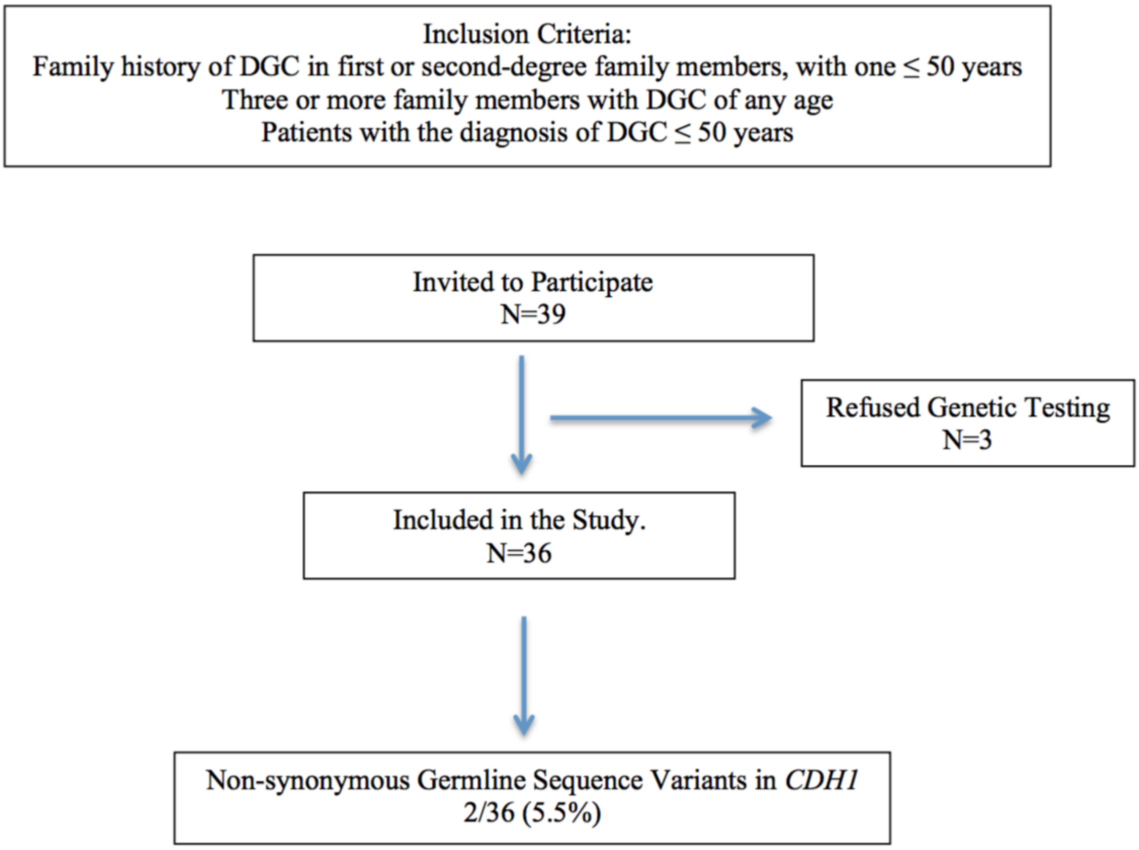

Figure S2: Flowchart of the study. DGC: Diffuse gastric cancer.
